# Comparison of Sinonasal Symptoms in Patients with Nasal Septal Deviation and Patients with Chronic Rhinosinusitis 

**Published:** 2013

**Authors:** Mohammad Naeimi, Maria Garkaz, Mohammad Reza Naeimi

**Affiliations:** 1 *Ear,Nose,Throat, Head and Neck Surgery Research Center, Mashhad University of Medical Sciences, Mashhad, Iran. *; 2 *Departmen of Otorhinolaryngology, Mashhad University of Medical Sciences, Mashhad, Iran.*; 3 *MedicalStudent Semmelweise Universit, Budapest, Hungary.*

**Keywords:** Chronic rhinosinusitis, Septal deviation, Symptoms of sinonasal disease

## Abstract

**Introduction:**

Disorders of the nose and paranasal sinuses are among the most common chronic illnesses. Although considerable progress has been made in the medical and surgical control of these diseases, a large number of questions relating to the diagnosis, evaluation, and treatment of these conditions remain unanswered. The aim of the present study was to evaluate differences in the frequency of symptoms and disease severity in patients with nasal septal deviation (NSD) compared with chronic rhinosinusitis (CRS).

**Materials and Methods:**

A total of 156 patients, divided into NSD and CRS groups, were studied in relation to symptoms and disease severity. Patients were selected from those referred to the Ear, Nose, and Throat (ENT) Wards of the Imam Reza and Ghaem Hospitals, who had not responded to a variety of treatments. Depending on the type of disease, patients were candidates for either septoplasty or endoscopic sinus surgery. The Rhinosinusitis Symptom Inventory was administered to measure the severity of symptoms, with scores assigned based on the answers given by patients (Likert scale). Scores were compared between the CRS and NSD groups.

**Results:**

A total of 156 patients (78 with NDS and 78 with CRS) entered the study in overall sinonasal symptoms were more prevalent in CRS group. Nasal congestion, runny nose, earache, toothache, and smelling disorder were significantly more common in the CRS group (P<0.001); while there were no significant differences in symptoms such as facial pressure, fever, or headache between the two groups (P>0.05).

**Conclusion:**

Patients with CRS manifested statistically significantly greater sinonasal symptom scores than patients with NSD.

## Introduction

Diseases of the nose and paranasal sinuses are among the most common chronic illnesses. Based on studies carried out in the United States, approximately thirty million people suffer from allergic rhinitis, chronic rhinitis, and/or chronic rhinosinusitis (1). Considerable progress has been made in the medical and surgical control of these conditions; however, a large number of questions relating to the diagnosis, evaluation, and treatment of the diseases remain unanswered. An evaluation of the symptoms of these diseases, as well as a comparison of their severity, would be valuable for diagnosis and for determining the appropriate course of treatment (use of antibiotics, antihistamines, or corticosteroids for example). The objective of this study was to compare sinonasal symptoms and disease severity between patients with nasal septal deviation (NSD) and patients with chronic rhinosinusitis (CRS). 

## Materials and Methods

This case-control study was performed in 2011 at the Mashhad University of Medical Sciences. Patients were selected from among those referred to the Ear, Nose, and Throat (ENT) Wards of the Imam Reza and Ghaem University Hospitals. A total of 156 partici- pating patients were divided into two equal groups. The first group was composed of 78 candidates for NSD surgery and the second group consisted of 78 patients planning to undergo endoscopic sinus surgery for CRS without nose septum deviation. All patients completed the Rhinosinusitis Symptom Inventory (RSI). Informed consent was obtained from all subjects prior to any investigation, and the protocol was approved by the Mashhad University of Medical Sciences Ethics Committee, Mashhad. 

A statistical comparison between the two groups was performed with regard to symptoms and severity. Sampling was performed using the non-probability convenience method. Data collection was performed by completing the RSI questionnaire. Data were analyzed statistically using SPSS software.

Descriptive indexes of the samples were determined after entering data into SPSS software. Chi-square, Mann-Whitney, Wil- coxon, and Kruskal-Wallis tests were used to analyze and evaluate the data. 

## Results

 A total of 96 male and 60 female patients were included in this study (P=0.149), with 52 and 44 male patients represented in the NSD and CRS groups, respectively (P=0.188). All patients were aged less than 70 years, with the majority aged between 20–30 or 30–40 years (70 and 40 patients, respectively) (P=0.001). A total of 105 cases out of 156 patients in the study had a positive family history of nasal and paranasal diseases. Twenty-four NSD and 62 CRS patients suffered from nasal congestion (P<0.001) (Table 1**).**

**Table 1:** Prevalence of sinonasal symptoms in patients

	NSD (N=78) n (%)	CRS (N=78) n (%)	Total (N=156) n (%)	P-value
Nasal congestion	24 (30.77)	62 (79.49)	86 (55.13)	<0.001
Smelling disorder	18 (23.08)	68 (87.18)	86 (55.13)	<0.001
Runny nose	19 (24.36)	59 (75.64)	78 (50.00)	<0.001
Nasal obstruction	56 (31.22)	50 (79.31)	106 (45.26)	0.340
Headache	33 (42.31)	30 (38.46)	63 (40.38)	0.143
Corticosteroid consumption	50 (64.10)	71 (91.30)	121 (77.56)	0.056
Antibiotic administration	55 (70.51)	75 (96.15)	130 (83.33)	0.079
Antihistamine consumption	67 (85.90)	59 (75.64)	126 (80.77)	0.476

**Fig 1:**
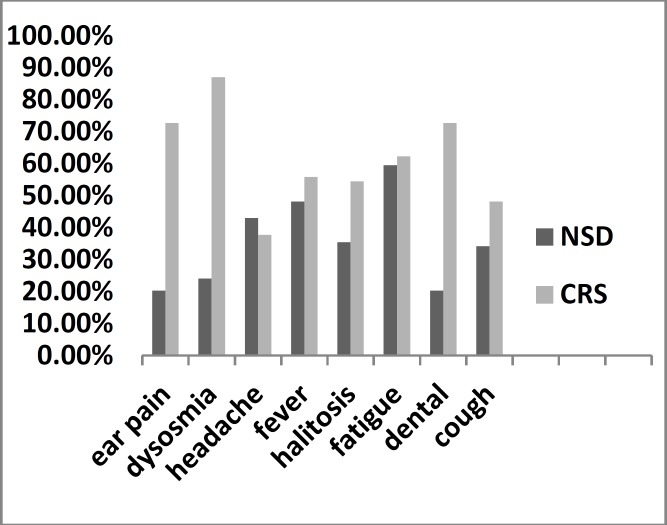
Number of patients reporting symptoms in NSD and CRS groups

More patients in the CRS group than in the NSD group reported symptoms in all categories except for headaches. Nasal congestion, runny nose, earache, toothache, and smelling disorder were significantly more common in the CRS group (P<0.001); while there were no significant differences in symptoms such as facial pressure, fever, or headache between the two groups (P>0.05) (Fig 1).

## Discussion

A total of 156 patients were examined in this study (78 in the NSD group and 78 in the CRS group). The two groups had a similar positive family background regarding sinonasal diseases.

CRSs are among the diseases which most frequently involve paranasal sinuses disorders (2,3). Thus, research into the diagnosis, pathophysiology, and treatment of this condition is important (1,4,5,6,7). 

In general CRS patients require a more intensive treatment in comparison with stand-alone NSD patients (3,8). For example, in the early stages of treatment and follow-up of CRS, a wide range of antibiotics is administered (3,8,9). Due to the difficulties of treatment, CRS is recognized as an infectious disease, and has a distinct pathophysiology from that of chronic NSD (10-12). For these reasons, CRS is considered a more severe and harmful disease in comparison with other nasal and paranasal sinus diseases, such as NSD or allergic rhinitis (3,13-15). Furthermore, upon close observation, there is a slight difference between the facial symptoms of the two groups of patients (3). There is also the possibility that the symptoms of the CRS disease return even after intensive medical treatment or after surgery (3,9). Thus, due to their wider range of disease symptoms, it can be concluded that CRS patients need more care and attention, as well as more intensive medical regimens. Intensive care and attention leads, inevitably, to a wider range of side effects and sequelae, including various disabilities, absences from work, and higher frequencies of doctor visits (3,5).

Upon seeking medical treatment, CRS patients tend to have more severe nasal symptoms in comparison with NSD-only patients. This difference is predominantly due to smelling disorders, which are highly evident in CRS patients (3). Similarly, CRS patients suffer from more severe nasal congestion and runny-nose symptoms than NSD-only patients (16,4).

Facial-pressure symptoms, as well as headaches, tend to suggest CRS diagnoses. Indeed, sometimes, facial symptoms alone can be used to diagnose and differentiate CRS patients from NSD patients (17). Headaches in this group of patients are found more often in the sinusal area, and can be diagnosed during examinations (1).

However, when the comparisons between the two groups in this study were based only on the facial symptoms of the patients, it would have been difficult to differentiate between the two groups. The reason behind the lack of difference in facial symptoms between the two groups is not clear. There is a possibility that NSD patients confuse headache with the symptoms of facial pressure or pain. Since the facial pain could be due to septum deviation, there are different opinions regarding this finding (1,3). The relationship between headache and CRS and NSD and even allergic rhinitis has been referred to in several studies. However, the patients in each group reported different degrees of headache (12). For instance, in a study carried out by Arunachalam et al, it was reported that 72 per cent of the patients undergoing septoplasty operation had a lesser degree of headache after the operation (11,3). A study by Harley et al stated similar results, also suggesting that headache in NSD patients was reduced after septoplasty (12,3). Another study referred to the advantages of sinus endoscopy on headaches in CRS patients (3,13).

Thus, symptoms such as facial pain or pressure could be detected in both groups of patients. Therefore, it can be concluded that the diagnosis of CRS should not be based only on symptoms identified by the patients, but also on the basis of other methods such as computed tomography (CT) scans and endoscopy (3,14).

In comparison with NSD patients, CRS patients have a longer history of using nasal steroids (3,4), while antihistamine use does not differ significantly between the two groups (1,3). Similarly, CRS patients tend to use antibiotics for a longer period of time (3).

Due to the higher rate of complaints in CRS patients, more intensive treatments have been used for treating sinunasal symptoms (3,13). Typically, due to mechanical issues, NSD patients are mistakenly treated with nasal steroids.

Previous studies in this field demonstrated that the annual follow-up and treatment expenses of CRS patients can average$1,000 per patient (3). Accounting for reduced productivity through lost working days raises the yearly expense to $1,600 per patient (3,8). In general, due to the more prominent symptoms of their disease in comparison with NSD patients, CRS patients are absent more frequently from work, and consume greater healthcare resources. Thus, according to the economic standards, CRS is a more costly disease compared with NSD (3).

## Conclusion

 In the context of previous studies, the results of this research showed that prevalence of sinonasal symptoms were higher in CRS patients than in patients with NSD. The treatment received by CRS patients was more intensive than that received by NSD patients; with patients spending more time under medical care, requiring increased frequency of doctor visits, and accruing more sick days.

There were no significant differences in symptoms regarding the gender of the patients. The majority of NSD patients were aged between 20–30 years, compared with CRS patients who were represented across almost all age groups.

Based these data and previous research, further studies need to be performed to investigate the pathophysiological factors of such disorders and also to identify new methods for diagnosis and differentiation of the two groups of patients.
